# Cruciferous Vegetable Intervention to Reduce the Risk of Cancer Recurrence in Non–Muscle-Invasive Bladder Cancer Survivors: Development Using a Systematic Process

**DOI:** 10.2196/32291

**Published:** 2022-02-15

**Authors:** Karen H Kim Yeary, Nikia Clark, Frances Saad-Harfouche, Deborah Erwin, Margaret Gates Kuliszewski, Qiang Li, Susan E McCann, Han Yu, Catherine Lincourt, Jamie Zoellner, Li Tang

**Affiliations:** 1 Cancer Prevention and Control Roswell Park Comprehensive Cancer Center Buffalo, NY United States; 2 New York State Cancer Registry New York State Department of Health Albany, NY United States; 3 Department of Epidemiology and Biostatistics University at Albany School of Public Health Albany, NY United States; 4 Department of Urology Roswell Park Comprehensive Cancer Center Buffalo, NY United States; 5 Department of Pharmacology & Therapeutics Roswell Park Comprehensive Cancer Center Buffalo, NY United States; 6 Department of Biostatistics and Bioinformatics Roswell Park Comprehensive Cancer Center Buffalo, NY United States; 7 Department of Public Health Science University of Virginia Charlottesville, VA United States

**Keywords:** non–muscle invasive bladder cancer survivors, dietary intervention, cruciferous vegetable, cancer survivorship, cancer recurrence

## Abstract

**Background:**

Bladder cancer is one of the top 10 most common cancers in the United States. Most bladder cancers (70%-80%) are diagnosed at early stages as non–muscle-invasive bladder cancer (NMIBC), which can be removed surgically. However, 50% to 80% of NMIBC cases recur within 5 years, and 15% to 30% progress with poor survival. Current treatments are limited and expensive. A wealth of preclinical and epidemiological evidence suggests that dietary isothiocyanates in cruciferous vegetables (Cruciferae) could be a novel, noninvasive, and cost-effective strategy to control NMIBC recurrence and progression.

**Objective:**

The aim of this study is to develop a scalable dietary intervention that increases isothiocyanate exposure through Cruciferae intake in NMIBC survivors.

**Methods:**

We worked with a community advisory board (N=8) to identify relevant factors, evidence-based behavior change techniques, and behavioral theory constructs used to increase Cruciferae intake in NMIBC survivors; use the PEN-3 Model focused on incorporating cultural factors salient to the group’s shared experiences to review the intervention components (eg, the saliency of behavioral messages); administer the revised intervention to community partners for their feedback; and refine the intervention.

**Results:**

We developed a multicomponent intervention for NMIBC survivors consisting of a magazine, tracking book, live telephone call script, and interactive voice messages. Entitled *POW-R Health: Power to Redefine Your Health*, the intervention incorporated findings from our adaptation process to ensure saliency to NMIBC survivors.

**Conclusions:**

This is the first evidence-based, theoretically grounded dietary intervention developed to reduce bladder cancer recurrence in NMIBC survivors using a systematic process for community adaptation. This study provides a model for others who aim to develop behavioral, community-relevant interventions for cancer prevention and control with the overall goal of wide-scale implementation and dissemination.

## Introduction

### Background

Bladder cancer is the sixth most common cancer in the United States, contributing to >80,000 new cases and 17,000 deaths annually [1]. Bladder cancer mostly affects older adults, with an average age of diagnosis of 73 years. Men are 4 times more likely than women to be diagnosed with the disease, and the incidence rates in White men are double those of Black men [2]. Most bladder cancers (70%-80%) are diagnosed at early stages as non–muscle-invasive bladder cancer (NMIBC) [3]. After surgical removal, NMIBC frequently recurs (50%-80%), with some patients experiencing multiple recurrences at similar stages and others (15%-30%) progressing to muscle-invasive disease, which is associated with cystectomy and poor survival [3-5]. Novel, noninvasive, and cost-effective strategies to control NMIBC recurrence and progression are urgently needed.

Dietary isothiocyanates (ITCs) are phytochemicals primarily derived from cruciferous vegetables (Cruciferae, eg, kale, turnips, and broccoli) with multifaceted anticancer mechanisms [6,7]. ITCs are particularly promising against bladder cancer given that orally ingested ITCs are rapidly concentrated in urine and delivered to the bladder, maximizing direct exposure [8,9]. A compelling body of preclinical [8,10-12] and epidemiological evidence [13,14] has demonstrated the important role of dietary ITCs and ITC-rich Cruciferae in preventing bladder cancer recurrence and progression by inhibiting the growth of bladder cancer cells and preventing tumor progression. However, the consumption of the primary dietary source of ITCs—Cruciferae—is generally low among NMIBC survivors, with studies reporting 0.44-0.45 servings per day [13,15] and urinary ITC levels at approximately 4.4 µM, which is below the average of 10 µM of ITCs observed to inhibit ≥50% of bladder cancer cell growth in in vitro models [8,10].

There are currently no national guidelines regarding optimal Cruciferae intake to prevent bladder cancer recurrence in NMIBC. Urinary ITC levels are affected by the following: (1) ITC yield varies up to 300-fold from raw Cruciferae [16], (2) cooking reduces ITC yield [16], and (3) peak urinary concentration is achieved within 3 hours of dosing and >50% of the dose is excreted and accumulated in urine within 8 hours of dosing [11,17]. On the basis of these findings, we hypothesize that at least one serving (~1 cup raw or ½ cup cooked) of Cruciferae per day, with guidance on the choice of vegetables and cooking conditions, and consumption of Cruciferae at dinner time to minimize ITC excretion from frequent urination during the day will increase urinary ITC levels to the desired doses needed to exert anticancer activities in the bladder.

### Objective

We describe a systematic process through which we developed an evidence-based Cruciferae intervention for NMIBC survivors with the goal of increasing Cruciferae intake in NMIBC survivors by at least one serving per day. The objectives of this study are to (1) describe how a well-known systematic process for evidence-based intervention adaptation can be used to develop a dietary intervention for cancer survivors and (2) detail an evidence-based, potentially scalable Cruciferae intervention that incorporates current preclinical and epidemiological data and NMIBC survivor perspectives. Our intention is that this study will facilitate future research that aims to improve NMIBC survivorship outcomes and inform the development process of future dietary interventions for cancer survivors.

## Methods and Results

### Overview

Consistent with the community-engaged approach of our study and other studies using a participatory process [18,19], we collaborated with an 8-member community advisory board to develop our intervention. We engaged our institution’s networks to select community advisory board members that represented clinic staff, clinic providers, clinical research advocates, and NMIBC survivors, including an Asian male clinical urologist, a White female urology nurse, a Black cancer research advocate, 4 White male NMIBC survivors, and a White female NMIBC survivor. The constitution of the community advisory board represented both the local population and the diversity of patients with bladder cancer in terms of race and gender. Together, we went through a four-stage adaptation process [20] that consisted of (1) information gathering, (2) preliminary adaptation design, (3) preliminary adaptation tests, and (4) adaptation refinement (Multimedia Appendix 1). In stage 1, we collected data to inform intervention development. In stage 2, we decided on the skeleton of the intervention based on the data from stage 1. In stage 3, we developed a preliminary draft of the intervention. In stage 4, we refined the intervention. In this section, we report the methods and results of each of the 4 stages. The Institutional Review Board at Roswell Park approved all materials and methods.

### Stage 1: Information Gathering

#### Stage 1 Methods

We collected data from the existing literature on dietary interventions for older adults, a discussion group with clinical staff, and in-depth interviews with NMIBC survivors.

##### Literature Review

The first author (KY) drew from the large body of literature on evidence-based behavioral fruit and vegetable interventions, focusing on interventions that significantly increased vegetable intake among participants whose demographic characteristics mirrored most NMIBC survivors (ie, aged ≥65 years and male) [21,22]. The search terms *dietary*, *diet*, *nutrition*, *vegetable*, *fruit*, *intervention*, and *review* were entered into PubMed to find review articles. Review articles were used to ascertain modalities (eg, telephone and in person) of intervention delivery that were associated with significant changes in dietary intake in older adults, and we chose the mode of intervention delivery based on this review. The first author then selected articles from the review papers that used our chosen intervention modality and caused significant changes in vegetable intake. From these studies, the first author, who is trained in qualitative methodology and behavioral interventions, identified the behavior change techniques incorporated into the interventions by using the behavior change technique taxonomy (version 1) [23], which identifies 93 distinct behavior change techniques. Behavioral theories used in these effective vegetable interventions were also examined to choose a theory to ground our intervention. After selecting the behavioral theory to ground the intervention, behavior change techniques that targeted the theory’s constructs were included in the intervention’s adaptation.

##### Discussion Group

After the literature review, we asked the clinical members of our community advisory board (ie, the urologist, nurse in urology clinic, and research advocate) to participate in a WebEx (Cisco Systems) discussion group to ascertain the factors necessary to maximize the saliency of an intervention for NMIBC survivors. Community members of the community advisory board (ie, NMIBC survivors) were not included in the discussion as we wanted to ascertain the shared experience of clinic members serving patients with NMIBC, which is a distinct experience from NMIBC survivors receiving treatment. During the discussion session, the facilitator (KY) asked discussion group members to draw from their experience working with NMIBC survivors to suggest topics to include in a Cruciferae intervention. The group was also asked what obstacles NMIBC survivors may have in eating more Cruciferae and about potential strategies to overcome any identified obstacles. The facilitator took notes during the discussion, summarized the main ideas from the discussion group, and recirculated the notes from the discussion to the discussion group members for verification.

##### In-depth Interviews

We then conducted in-depth interviews with NMIBC survivors. We used the PEN-3 Model [24,25] to develop the interview guide and analyze the data. The PEN-3 Model focuses on incorporating cultural factors salient to groups that share a similar collection of experiences [26,27]. Given that NMIBC survivors share a unique cancer treatment and control experience, we decided to use the PEN-3 Model to ensure that the intervention was salient to them (Multimedia Appendix 2) [24,25]. Analysis of the data consisted first of open coding followed by categorization of the codes based on PEN-3 dimensions.

We conducted in-depth interviews over the telephone with NMIBC survivor members of our community advisory board using a semistructured interview guide. The guide asked the participants to describe their experience with bladder cancer, their health goals and priorities, their knowledge of Cruciferae intake and cancer risk, barriers to and facilitators of higher Cruciferae intake, their food shopping habits, their preparation and planning of meals, and their social support for vegetable intake. All interviews were audio-recorded and transcribed.

A total of 2 research team members (KY and DE) began the PEN-3 process using an open coding technique in accordance with standard qualitative methodology [28,29]. Each research team member independently identified initial patterns, themes, and codes. For the *Cultural Identity* domain, the PEN dimensions of *Person*, *Extended Family*, and *Neighborhood* were considered in developing the codes. The 2 research team members then used the constant comparison approach to agree upon a list of final codes that emerged from the in-depth interview data. The finalized code list was then categorized by the dimensions of the *Relationships and Expectations* domain as *Perceptions*, *Enablers*, and *Nurturers.* We then used the *Cultural Empowerment* domain to determine whether the perceptions, enablers, and nurturers were *Positive*, *Existential*, or *Negative*. Any differences in categorization were resolved through discussion. The research team then used the analyzed data to develop intervention strategies.

#### Stage 1 Results

##### Literature Review

There were 6 review articles on dietary change interventions [30-35], of which 2 (33%) focused on dietary change in older adults [30,31]. The reviews reported that both face-to-face and telephone-based interventions had proven efficacy in changing dietary behavior in older adults [31,36]. Thus, to maximize our intervention’s potential for scalability, we decided that our intervention would be telephone-based.

Of the 6 review articles, 4 (67%) used a telephone-based intervention and reported significant changes in vegetable intake in older adults (aged ≥60 years) [21,22,37,38]. The first author (KY) reviewed these 4 successful interventions and identified the application of the following evidence-based behavior change techniques [23]: problem solving or coping planning, social support (practical, general, and emotional), goal setting (outcome and behavior), prompts or cues, and feedback on behavior.

The 4 successful vegetable interventions reported the use of the transtheroretical model, health belief model, or social cognitive theory, or no theory at all [30,37]. There was no consensus regarding the intervention dosage or frequency of contact by intervention modality (eg, number of calls needed for dietary change).

##### Discussion Group

The discussion group included all clinical members of the community advisory board (3/8, 38%) and lasted approximately 60 minutes. Discussion group members reported that some of their patients with NMIBC called their bladder cancer a “fake cancer” because of their perception that it was a “just on the surface” cancer that had been successfully treated. These patients with NMIBC did not have a detailed understanding of how their cancer may progress and perceived the physician “scraping them” regularly as sufficient for long-term treatment. The NMIBC itself did not involve intensive treatment regimens such as chemotherapy or radiation therapy and, thus, was not seen as a dangerous disease. Consequently, discussion group members stated that the intervention needed to help the patient take NMIBC seriously without scaring them—that even though the risk of dying is low, the goal of the NMIBC survivor should be to prevent recurrence and save their bladder.

Discussion group members also listed other potential obstacles to NMIBC survivors changing their dietary habits, including access to and cost of fresh Cruciferae, the intervention bringing back negative childhood memories of having to “eat their vegetables,” and the physical discomfort (via gas) that could be caused by consuming Cruciferae. Despite the potential obstacles identified, discussion group participants stated that many of their patients asked them what they should eat and were eager to make dietary alterations to help fight the disease. They recommended ascertaining who did the grocery shopping for the NMIBC survivor as most survivors are older men who may not do their own food shopping.

##### In-depth Interviews

Project staff conducted 4 interviews with community advisory board members who were NMIBC survivors. Each interview lasted approximately 45 minutes. The NMIBC survivors included 3 non-Hispanic White men (3/4, 75%) and 1 non-Hispanic White woman (1/4, 25%). The mean age was 73 (SD 8.9) years, with 3 of them being married (3/4, 75%) and 1 retired (1/4, 25%). Of the 4 participants, 2 (50%) reported completion of high school as their highest level of education, whereas 1 (25%) reported at least some college education, and 1 (25%) reported postgraduate education. All participants (4/4, 100%) had health insurance, with 3 reporting Medicare (3/4, 75%) and 1 reporting Medicaid (1/4, 25%). Multimedia Appendix 3 presents the results of the PEN-3 analysis according to the *Relationships and Expectations* domain of perceptions, enablers, and nurturers. Some of the codes are listed more than once if they covered >1 PEN-3 dimension. Within each domain, data were then categorized by the *Cultural Empowerment* domain dimensions of positive, existential, and negative.

##### Perceptions

###### Positive

NMIBC survivors’ positive perceptions included knowledge regarding the benefits of healthy eating, particularly vegetable intake. Some participants spoke about the daily routine of having no choice but to eat what was on their plate—including fresh vegetables—in the “previous generation” when they were children. NMIBC survivors believed that fresh vegetables were best and that information from physicians and others in authority had more validity.

###### Existential

Many NMIBC survivors described their initial diagnosis of bladder cancer as a shock. Some described no symptoms before diagnosis and described the treatment as minimal. Many participants also said they were unaware of the evidence linking Cruciferae with decreased bladder cancer recurrence.

###### Negative

Negative perceptions included seeing bladder cancer as a “lesser” or “good” cancer and the sufficiency of maintenance medical visits to prevent bladder cancer recurrence. Many NMIBC survivors said they felt well and wanted to maintain a positive attitude; consequently, they were unaware of the high risk of recurrence of bladder cancer. Regarding the consumption of Cruciferae, participants reported the flavor, texture, appearance, and effort required for preparation as barriers. Overcoming habits of poorer eating choices was also a negative perception.

##### Enablers

###### Positive

All the NMIBC survivors emphasized the wife’s strong role in promoting healthy dietary behaviors in survivors. Other positive enablers included perceived easy access to a variety of fresh vegetables and vegetable recipes. Many survivors reported that their bladder cancer diagnosis was a catalyst for eating healthier.

###### Existential

Some NMIBC survivors described difficulty in acknowledging that they were cancer survivors as their cancer treatment resulted in minimal side effects.

###### Negative

Negative enablers included feelings of guilt for being considered a cancer survivor because of the less intensive treatments they underwent compared with other cancer survivors. Consequently, some did not consider bladder cancer a “real” cancer for them to be vigilant in preventing. The time and effort required to prepare Cruciferae, friends and fellow survivors’ poor dietary habits, and television advertisements for less healthy food choices were other identified negative enablers. The medical system’s provision of ongoing monitoring after treatment also served as a negative enabler for dietary change as medical management alone was perceived as enough to prevent recurrence.

##### Nurturers

###### Positive

NMIBC survivors overwhelmingly discussed the survivor’s wife as a positive nurturer who set nutritional priorities, prepared meals, and went grocery shopping. Physicians were also identified as positive nurturers to convince survivors of the importance of Cruciferae.

###### Existential

There were no existential nurturers identified.

###### Negative

Negative nurturers included having family members with poor health who required care and took away from the survivors’ ability to care for themselves and the lack of a wife, which would make healthy food preparation tasks insurmountable to the survivor.

### Stage 2: Preliminary Adaptation Design

#### Stage 2 Methods

We performed an iterative process to incorporate the results from stage 1 to develop preliminary drafts of our Cruciferae intervention for NMIBC survivors. The results from stage 1 were discussed as a research team and with our community advisory board. After we came to a consensus concerning how to include the results from stage 1, a subgroup within our team developed an initial draft of the intervention, which was then recirculated back to the larger group for feedback and subsequent refinement.

#### Stage 2 Results

##### Literature Review

Data from stage 1 were used to develop the basic framework for our intervention. We decided to incorporate all the evidence-based behavior change techniques [23] used by the 4 papers [21,22,37,38] that described phone-based interventions significantly increasing vegetable intake in older adults: problem solving or coping planning, social support (practical, general, and emotional), goal setting (outcome and behavior), prompts or cues, and feedback on behavior. We incorporated these behavior change techniques throughout the intervention, for example, we included an action plan for each participant to complete to set short- and long-term Cruciferae intake goals (goal setting) and a process for the participants to receive ongoing feedback regarding their Cruciferae intake through interactive voice response (IVR), which is described below (feedback on behavior). Given that dietary interventions grounded in theory may be more effective than non–theoretically-based interventions [30], we decided to base our intervention on theory. We chose social cognitive theory as the evidence-based behavior change techniques identified in our review were linked to 8 of the 11 major social cognitive theory constructs (self-efficacy; outcome expectations; knowledge; social support; barriers and opportunities; behavioral skills; intentions; and reinforcement and punishments). Thus, these constructs were interwoven throughout our intervention (eg, intervention components were designed to boost self-efficacy, knowledge, and specific behavioral skills by incorporating information about Cruciferae and strategies to increase intake). The 3 major constructs of social cognitive theory that were not used in the 4 studies reviewed (collective efficacy, observational learning, and normative beliefs) were included by adding behavior change techniques (restructuring the social environment, credible source, and social comparison) that reflected these constructs.

We chose a telephone-based modality for intervention delivery as this method has proven efficacy in changing dietary behavior in older adults [31] and would arguably be more feasible to implement on a wider scale compared with face-to-face interventions [36]. We also decided to deliver the bulk of our telephone calls through automated calls or IVR telephone messages to facilitate future scale-up. Given the lack of consensus regarding intervention dosage or frequency of contact by intervention modality, we decided to include 1 mailing, 1 live telephone call, and 11 IVR calls over a 6-month period. We adapted an IVR template with proven success in a previous dietary change intervention [39] to develop our IVR calls.

Thus, the structure of our intervention consisted of (1) an initial mailing of an informational magazine and booklets to track Cruciferae intake; (2) a follow-up telephone call with research staff to verify understanding of the educational information, provide instructions on how to use the track books, and help participants complete a personalized action plan whereby participants identified barriers and facilitators to meet identified goals; and (3) 11 IVR telephone calls whereby participants entered the amount of Cruciferae consumed and received tailored feedback based on their reported consumption.

Specific content within our intervention was informed through the results of the discussion group and in-depth interviews conducted in stage 1.

##### Discussion Group

We specifically addressed the idea that NMIBC is perceived as a fake cancer by emphasizing the importance of taking the cancer seriously. Potential obstacles to Cruciferae intake identified by the discussion group were also included in the IVR specifications. Identifying who in the household did the grocery shopping and ensuring that they were engaged in the participant’s action plan was also included.

##### In-depth Interviews

We developed intervention strategies based on the categories of the PEN-3 framework according to which the qualitative data were sorted. Specifically, the intervention strategies were based on the perception, enabling, and nurturing dimension within the *Relationships and Expectations* domain. Intervention strategies either reinforced factors that supported increased Cruciferae intake or revised factors that discouraged dietary change to facilitators of dietary change. The specific strategies, sorted by reinforcing or revised strategies, are presented in Multimedia Appendix 4. Overall, the strategies emphasized the high recurrence rate among NMIBC survivors and the fact that medical monitoring alone will not prevent recurrence. Identified strategies included presenting information about significant relationships between Cruciferae intake and bladder cancer etiology, the role of Cruciferae in staying strong and living long, easy ways to prepare Cruciferae, and an emphasis on fresh vegetable consumption. Engaging spouses or partners, family, and friends to change their dietary behavior was also promoted. Given the survivors’ lack of saliency with the word *cruciferous*, the intervention was branded as the *Power to Redefine Your Health* (POW-R Health) Program, wherein Cruciferae are referred to as *power* vegetables. The POW-R Health Program is promoted as an intervention developed and endorsed by medical professionals.

### Stage 3: Preliminary Adaptation Tests

#### Stage 3 Methods

On the basis of the data collected in stages 1 and 2, we developed draft educational materials, a script for the live call, and the IVR specifications. Our team then engaged in an iterative process to ensure that the intervention was salient to NMIBC survivors. The initial drafts of the magazine, track books, and IVR specifications were reviewed by the team. The team interventionist pilot-tested the live call script with 3 NMIBC survivors in the community advisory board.

#### Stage 3 Results

Team members suggested that a project logo be developed to promote continuity between the various pieces of the intervention. Thus, we worked with the NMIBC survivors in the community advisory board to develop a project logo. NMIBC survivors reviewed various logos and chose a circular one with pictures of Cruciferae. The logo was included in the educational materials (magazine and track book).

Some team members reported that directly stating in the intervention materials that 50% to 80% of NMIBC recurs within 5 years may scare survivors and recommended removing the statistic. Thus, we developed different versions to convey the high risk of bladder cancer recurrence and presented these versions to the NMIBC survivors in the community advisory board. The NMIBC survivors agreed that the high risk of recurrence needed to be directly conveyed by reporting the statistic. Several NMIBC survivors stated that “telling the truth as it is” would not unnecessarily scare survivors but rather emphasize the urgency to care for one’s health.

A larger font size (14 points) was recommended to make the magazine easier to read in addition to pictures showing diverse older adults. The team decided to add to the track book cover the total cups of Cruciferae consumed throughout the week to facilitate participant entry of that information for the IVR calls. The community advisory board thought that the track book was easy to understand and use.

All recommended that the initial 60-minute telephone call be shortened; thus, the initial portion of the call that included an in-depth overview of the magazine was changed from being delivered to every participant to being delivered only to those participants who did not review the magazine before the telephone call. The NMIBC survivors emphasized the importance of delivering the live call with energy and in a conversational manner.

Finally, the team recommended the creation of a magnet to remind the participants of the project IVR telephone number. In addition, the IVR number was highlighted at the end of the magazine, on the back page of the track book, and at the end of the live call to facilitate IVR use.

### Stage 4: Adaptation Refinement

#### Stage 4 Methods

Data from stages 1-3 were used to develop the near-final versions of the POW-R Health intervention, with these versions reviewed by the entire community advisory board and investigative team before finalization.

#### Stage 4 Results

Minor editorial adjustments were made to yield the final product—a Cruciferae intervention for NMIBC survivors designed to increase Cruciferae intake by at least one serving a day to reduce the risk of bladder cancer recurrence. The intervention consists of (1) an 11-page, 21.6 cm by 27.9 cm color magazine that includes information about the high risk of bladder cancer recurrence in NMIBC survivors, what Cruciferae are, and strategies to maximize ITC yield from Cruciferae consumption (≥1 cup a day at or after dinner, eaten raw or lightly cooked, of select high– to medium–ITC-yield Cruciferae) as well as an action plan with short- and long-term goal setting, identified obstacles, and strategies to meet set goals; (2) weekly track books to monitor Cruciferae intake throughout the duration of the 6-month intervention to facilitate self-monitoring; (3) a 45-minute live telephone call following receipt of the magazine and track books with an interventionist who ensures that the participants understand the materials sent, completes the action plan with the participants, and practices how to fill out the track books; and (4) 11 IVR calls after the live call spread across the remainder of the 6-month intervention whereby the participants enter the amount of Cruciferae consumed in cups and the IVR provides feedback based on the participants’ previously stated goals (eg, *Mark, I am really pleased to see that you accomplished your goal for the week! Congratulations on your success! The changes you are making are really going to help with your health in the long run*...or *Frank, based on what you ate in total last week, it looks like you didn’t quite hit your goal, but you did make some progress so congratulations on your success! Lots of people struggle with trying to eat more power vegetables*...). IVR calls are made automatically to the participants, and the participants can call into the IVR system if they miss an IVR call. A magnet with the project logo and IVR telephone number is given to the participants to maximize their IVR use.

## Discussion

### Principal Findings

We used a systematic approach to integrate the most up-to-date scientific knowledge of bladder cancer etiology, evidence-based behavior change techniques in dietary interventions, behavioral theory, and community expertise to develop POW-R Health. We believe that this systematic approach will maximize the intervention’s potential to produce clinically meaningful change [40]. We sought to produce an intervention salient to NMIBC survivors by making NMIBC survivors’ experiences, perceptions, behaviors, and knowledge the focal point of our process. Our choice of using telephone-based modalities and minimal staff contact also arguably increases the intervention’s scalability potential.

Compared with the development process of other dietary interventions for cancer survivors, our systematic approach for developing POW-R Health involves greater community engagement (eg, via the community advisory board) and the inclusion of more informational sources to inform intervention development. Most dietary interventions for cancer survivors have been developed without community input and created solely by the academic or clinical team [41,42]. Among the few that engaged the community, focus groups and surveys were used to garner feedback [43-46]. A total of 3 interventions used components from previous evidence-based interventions to develop their intervention [47-49]. Similar to our process, most published dietary interventions for cancer survivors were grounded in behavioral theory [41,42]. One study mirrored our process of including a literature review and choosing behavior change techniques to develop their intervention [43].

This study is not without limitations. First, members of the community advisory board did not include racially and ethnically diverse NMIBC survivors, although we attempted to mitigate this to some degree by including a diverse clinical team, among them a Black community cancer research advocate and cancer survivor. The resulting intervention model is expected to match well with the demographics of most NMIBC survivors and can provide a basis for further demographic tailoring in additional versions. Second, our literature review did not include multiple reviewers, which would have increased the rigor of the methods. Third, although many recurring themes emerged from the in-depth interviews with NMIBC survivors, we acknowledge that complete data saturation may not have been achieved. Acknowledging these limitations, we plan to conduct in-depth exit interviews at the end of the intervention study with all the participants to ascertain relevant aspects from more NMIBC survivors. These limitations should be considered within the strengths of this study, including collaboration with a community advisory board to guide all stages of this systematic intervention adaptation process and the first known evidence-based dietary intervention developed specifically for NMIBC survivors to reduce bladder cancer recurrence.

Currently, POW-R Health is being compared with an alternative treatment control in a pilot randomized controlled trial with NMIBC survivors. We hypothesize that, compared to the control, POW-R Health will significantly increase Cruciferae intake and urinary ITC levels and alter gene expression associated with bladder cancer recurrence. For cancer researchers who aim to develop behavioral interventions for cancer survivors, we encourage the use of similar systematic evidence-based frameworks that meaningfully engage communities in addition to evidence-based behavior change techniques and behavioral theory.

### Conclusions

Bladder cancer is a serious disease in which up to 80% of NMIBC survivors experience recurrence. Most patients with NMIBC have no treatment offered to prevent recurrence, instead relying on costly and frequent surveillance to monitor disease status with the goal of capturing recurrence or progression at early stages of treatment. Our intervention offers the potential for a promising, feasible, and accessible way for all NMIBC survivors to reduce bladder cancer recurrence. Our hope is that, if POW-R Health is proven to be efficacious, Cruciferae intake recommendations will become a part of standard clinical practice and meaningfully reduce negative outcomes associated with NMIBC.

## Appendix

Multimedia Appendix 1 The 4-stage adaptation process for intervention development.

Multimedia Appendix 2 PEN-3 Model.

Multimedia Appendix 3 Summary of qualitative results according to the PEN-3 framework.

Multimedia Appendix 4 Intervention strategies categorized by PEN-3 dimensions.

## Abbreviations

ITC: isothiocyanate
IVR: interactive voice response
NMIBC: non–muscle-invasive bladder cancer
POW-R Health: *Power to Redefine Your Health*
