# Supplementary material for: Cruciferous Vegetable Intervention to Reduce the Risk of Cancer Recurrence in Non–Muscle-Invasive Bladder Cancer Survivors: Development Using a Systematic Process
Source: JMIR Cancer. 2022 Feb 15;8(1):e32291. doi: 10.2196/32291 (PMC8889476; doi:10.2196/32291)
Supplement: Multimedia Appendix 1 [file cancer_v8i1e32291_app1.pptx]

## Slide 1
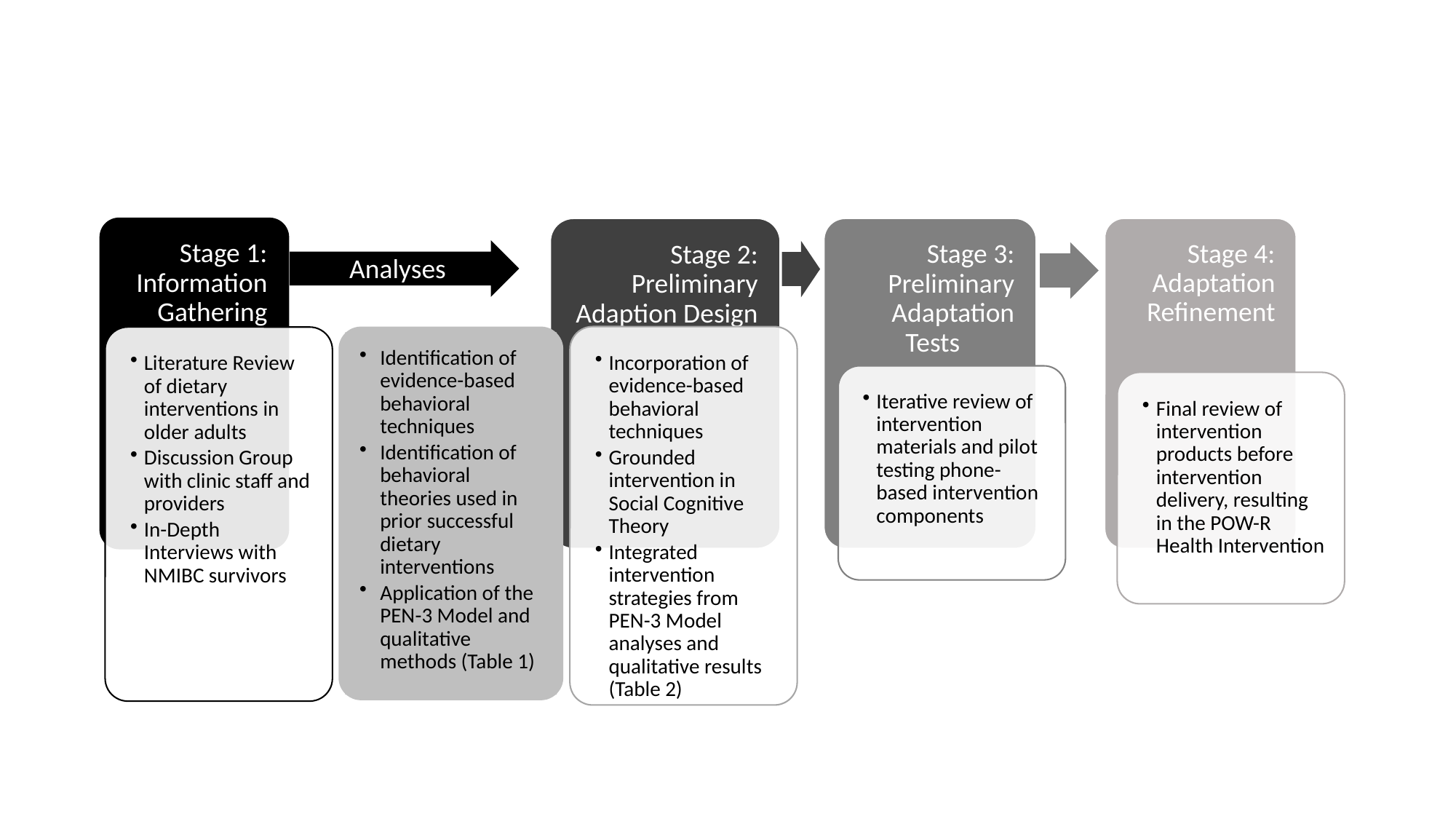

Analyses
Identification of evidence-based behavioral techniques
Identification of behavioral theories used in prior successful dietary interventions
Application of the PEN-3 Model and qualitative methods (Table 1)
