# Supplementary material for: Cruciferous Vegetable Intervention to Reduce the Risk of Cancer Recurrence in Non–Muscle-Invasive Bladder Cancer Survivors: Development Using a Systematic Process
Source: JMIR Cancer. 2022 Feb 15;8(1):e32291. doi: 10.2196/32291 (PMC8889476; doi:10.2196/32291)
Supplement: Multimedia Appendix 2 [file cancer_v8i1e32291_app2.docx]

The PEN-3 model focuses on the central role of culture to develop interventions that build upon identified cultural strengths. The model consists of three interlocking domains: Cultural Identity, Relationships and Expectations, and Cultural Empowerment. Within each domain are three factors that make up the acronym PEN. The Cultural Identity domain focuses on the role of the individual (*P*erson), social network (*E*xtended Family), and the community context (*N*eighborhood) in health behavior. The Relationships and Expectations domain assesses how an individual’s knowledge, attitude, and beliefs (*P*erception), community and structural factors (*E*nablers), and an individual’s social network context (*N*urturers) affect their health behavior. The Cultural Empowerment domain identifies positive (*P*ositive) factors, factors unique to the culture (*E*xistential) and negative factors (*N*egative) that influence health behavior.
