# Supplementary material for: Cruciferous Vegetable Intervention to Reduce the Risk of Cancer Recurrence in Non–Muscle-Invasive Bladder Cancer Survivors: Development Using a Systematic Process
Source: JMIR Cancer. 2022 Feb 15;8(1):e32291. doi: 10.2196/32291 (PMC8889476; doi:10.2196/32291)
Supplement: Multimedia Appendix 3 [file cancer_v8i1e32291_app3.docx]

|  | Positive (impact on risk reduction and eating Cruciferae) | Existential (neutral impact/exotic) | Negative (impact on risk reduction and eating Cruciferae) |
| --- | --- | --- | --- |
| Perceptions (individual attitudes, values, and beliefs) | - Worried about cancer coming back - “Losing weight and not eating crap and eating vegetables” is a way to lower bladder cancer recurrence - Being healthy is not being overweight - Prepare vegetables in different ways - Vegetables give them bowel movements - Emotions surrounding cancer - Fresh vegetables (homemade or homegrown) are best, not canned - Availability of fresh vegetables - “Can’t eat vegetables before bed, but at dinner is okay” - “I’m not going to sit around and get old” - “Bladder cancer changed my eating habits” - There is no good cancer - Link between cruciferous vegetables and bladder cancer makes sense, not surprising - Healthy eating prevents cancer, eating vegetables makes them strong and helps them live longer - Healthy eating means no fast food, no grazing or snacking, not much meat, eating vegetables and roughage - Doctors can convince patients to eat cruciferous vegetables (“I will listen to a doctor more than friends, family, or the media”); will listen to people in the field with more authority - “You cheated cancer once, don’t push it twice” - Eating vegetables was part of daily routine back in previous generation; “we had to eat vegetables and we didn’t have a choice” - Previous generation has respect for medical authorities more than the current generation | - “I’m always aware of my body and I had no idea I had cancer” - Did not know about link between cruciferous vegetables and bladder cancer - Hard to say cruciferous—need to come up with a different name - “I don’t feel like I had cancer” | - Positive attitude about cancer not coming back - Bladder washing and other medical treatments will prevent bladder cancer recurrence - Bladder cancer as a lesser cancer; seen by others as a good cancer - “I feel great and bladder cancer hasn’t altered my life” - Hard to say cruciferous—need to come up with a different name - Flavor, bitterness, texture, taste, and appearance - “Going back to my old ways” - Quality of life with ice cream versus quantity of life without ice cream (quality is more important) - “I’ve had a good life so no need to try too hard to lengthen it” - Guilt about their experience compared with other cancer survivors - “Can’t complain about my cancer” because family members have other cancer or poorer health - Taking a vitamin is as good as eating the real thing - “I don’t care, I’m going to eat what I’m going to eat” - Will only eat fresh vegetables - Healthy eating means no fast food, no grazing or snacking, not much meat, eating vegetables and roughage - “Can’t eat vegetables before bed, but at dinner is okay” - Emotions surrounding cancer - Takes too much time to prepare vegetables, too much effort - Health was not a priority in previous generation; cooking with lard and fat and large portions in previous generation set bad precedent - Men without wives have poorer health; would not eat healthy without a wife - Being healthy is not being overweight; “cheating [eating poorly] is okay from time to time as long as my weight is under control” |
| Enablers (societal or structural influences) | - Access, availability, and variety of vegetables in contrast to previous generation - Freezer full of steamers - Equipment in home to prepare vegetables in different ways - Keep putting vegetables in front of them - Recipes and menus with vegetables - Restaurants with healthy foods - Eating vegetables was part of daily routine back in previous generation; “we had to eat vegetables and we didn’t have a choice” - In previous generation, they ate what was put on the table - Homegrown or homemade vegetables, not canned foods, are best - Family exchanges food with each other - “Bladder cancer changed my eating habits” - Wife has a lot of influence on husband’s eating habits - Friends eat healthier, fellow cancer survivors become healthier (lose weight) - Support from the medical community for lifestyle change - Will listen more to doctor or some other medical authority than to media, friends, family, or others - Previous generation has respect for medical authorities more than current generation - Being a good example to their kids - Information about the benefits of cruciferous vegetables and bladder cancer - “Have information in my face” - Emotions following bladder cancer checkup | - “Don’t feel like I had cancer” | - Bladder washing and other medical treatments will prevent bladder cancer recurrence - Lack of availability of fresh vegetables - Never ate vegetables - Takes too much time to prepare vegetables, too much effort - Guilt about their experience compared with other cancer survivors - Family exchanges food with each other - Fellow cancer survivors and friends do not eat healthy - Health was not a priority in previous generation; cooking with lard and fat and large portions in previous generation set bad precedent; vegetables not a priority; a large amount of meat and potatoes - Homegrown or homemade vegetables, not canned foods, are best - Advertising on television about unhealthy foods - Men without wives have poorer health; without a wife, they would not take time to prepare vegetables, too much effort; would not eat healthy - Will listen more to doctor or some other medical authority than to media, friends, family, or others |
| Nurturers (supportive or discouraging influences from significant others) | - Keep putting vegetables in front of them - “Have information in my face” - Being a good example to their kids - Wife influences husband to eat healthier - Doctors need to convince patients that this is important |  | - Without a wife, husbands would not eat healthy - Preventing bladder cancer is not a priority if others in the family have poorer health |
