# Supplementary material for: Cruciferous Vegetable Intervention to Reduce the Risk of Cancer Recurrence in Non–Muscle-Invasive Bladder Cancer Survivors: Development Using a Systematic Process
Source: JMIR Cancer. 2022 Feb 15;8(1):e32291. doi: 10.2196/32291 (PMC8889476; doi:10.2196/32291)
Supplement: Multimedia Appendix 4 [file cancer_v8i1e32291_app4.docx]

| Dimensions | Reinforced by the intervention | Revised through the intervention |
| --- | --- | --- |
| Perceptions | - Cruciferous vegetables keep them regular (positive) - Cruciferous vegetables can be part of a healthy diet, help them have a healthy weight and live a long and healthy life (positive) - Prepare vegetables in different ways to eat cruciferous vegetables (positive) - Emphasize that fresh cruciferous vegetables have highest ITC^a^ yield and to not use canned cruciferous vegetables (positive) - Acknowledge availability of cruciferous vegetables (positive) - Note that cruciferous vegetables can be eaten at dinner (positive) - Eating cruciferous vegetables is consistent with not sitting around and getting old (positive) - Note that they probably are already eating vegetables, especially since initial diagnosis (positive) - Note that some may not find the link between cruciferous vegetables and cancer surprising (positive) - Reinforce current beliefs about healthy eating (positive) - Acknowledge that there is no good cancer, and cruciferous vegetables are possibly a way to keep bladder cancer from coming back (positive) - Reinforce previous generation’s eating habits—eating vegetables on a regular basis (positive) - Acknowledge that bladder cancer was in them and they were unaware of it, so it is important to be vigilant to prevent it even if they feel well (existential) - Target wife or spouse or partner in intervention materials; engage wife to eat cruciferous vegetables (positive) | - Acknowledge worry about cancer coming back and say how cruciferous vegetables may help stop cancer from coming back (positive) - Acknowledge beliefs about how to lower bladder cancer recurrence and emphasize the role of cruciferous vegetables (positive) - Talk about the best cooking methods to maximize ITC yield (positive) - Make sure materials state that they have been reviewed and endorsed by doctors (positive) - Educate about link between cruciferous vegetables and bladder cancer (existential) - Come up with easy/catchy name for project and cruciferous vegetables (*power* vegetables?; existential) - Provide information about the high chance of bladder cancer recurrence (negative) - Bladder washing and medical treatments alone will not prevent bladder cancer recurrence (negative) - Acknowledge that bladder cancer may be seen as a lesser or good cancer and may not have affected their life; however, it is still cancer and important to be prevented so they can remain healthy (negative) - Acknowledge that some cruciferous vegetables can have a bad taste, texture, or appearance; encourage different cooking methods and incorporation of cruciferous vegetables into different dishes (negative) - Acknowledge that changing old ways or old habits is hard and that they just want to eat what they want to eat (negative) - Acknowledge that they can want to eat less healthy foods, but they can still improve their diet to reduce bladder cancer risk and still enjoy many things in their current diet (negative) - Talk about how taking vitamins cannot replace cruciferous vegetables (negative) - Frozen vegetables are a good alternative to fresh vegetables (negative) - Provide recipes with quick and easy ways to prepare cruciferous vegetables (negative) - Important to eat cruciferous vegetables even if their weight is under control; having a healthy weight alone will not prevent bladder cancer recurrence (negative) - Acknowledge guilt and emphasize importance of taking care of oneself (negative) |
| Enablers | - Great variety and access to cruciferous vegetables (positive) - Can store frozen cruciferous vegetables in the freezer (positive) - Use cooking equipment to prepare vegetables in different ways (positive) - Acknowledge that, for some, eating vegetables is a continuation of how it was when they were younger (positive) - Talk about how fresh vegetables have the highest ITC yield; consistent with homegrown, fresh food back in the old days (positive) - Engage wife/spouse/partner to eat cruciferous vegetables (positive) - Acknowledge that cancer may have already changed their eating habits, so eating cruciferous vegetables is consistent with what they are already doing (positive) - Make sure materials mention that they are doctor-created and approved (positive) - Being a good example to their children and family (positive) - This program will build on current support from their medical community to have lifestyle changes (positive) - Have them ask family members to give them cruciferous vegetables for their health (positive) - Ask friends to support their efforts to eat cruciferous vegetables (positive) - Acknowledge that bladder cancer was in them and they were unaware of it, so it is important to be vigilant to prevent it even if they feel well (existential) | - Have cruciferous vegetables visible in the home (positive) - Include recipes and menus with cruciferous vegetables (positive) - Provide information about cruciferous vegetables and bladder cancer risk of recurrence (positive) - Bladder washing and medical treatments alone will not prevent bladder cancer recurrence (negative) - Frozen vegetables have good ITC yield that is comparable with fresh vegetables (negative) - Suggest low-effort, quick ways to prepare cruciferous vegetables (negative) - Suggest trying cruciferous vegetables (negative) - Acknowledge guilt and emphasize importance of taking care of oneself (negative) - Have them ask family members to give them cruciferous vegetables for their health (negative) - Acknowledge negative advertising on television (negative) - Make sure materials mention that they are doctor-approved (negative) - Eating cruciferous vegetables is a way to stay young (negative) - Present low-effort ways to prepare cruciferous vegetables (negative) |
| Nurturers | - Engage wife in implementing the intervention; ask husbands to share the intervention with their wives so they can help them and make sure their husbands do it (positive) - Make clear that the intervention is endorsed by medical professionals (positive) - Being a good example to their children and family by eating healthy (positive) - Make vegetables visible in the home (positive) - Have the person who shops for food buy the cruciferous vegetables they need (positive) | - Present low-effort ways to prepare cruciferous vegetables (negative) - Acknowledge guilt and emphasize importance of taking care of oneself (negative) |
